# All-inorganic cesium lead iodide perovskite solar cells with stabilized efficiency beyond 15%

**DOI:** 10.1038/s41467-018-06915-6

**Published:** 2018-10-31

**Authors:** Kang Wang, Zhiwen Jin, Lei Liang, Hui Bian, Dongliang Bai, Haoran Wang, Jingru Zhang, Qian Wang, Shengzhong Liu

**Affiliations:** 1 Key Laboratory of Applied Surface and Colloid Chemistry, Ministry of Education, Shaanxi Key Laboratory for Advanced Energy Devices, Shaanxi Engineering Lab for Advanced Energy Technology, School of Materials Science & Engineering, Shaanxi Normal University, 710119 Xi’an, People’s Republic of China; 2 School of Physical Science and Technology & Key Laboratory for Magnetism and Magnetic Materials of MoE, Lanzhou University, Lanzhou, 730000 People’s Republic of China; 3 Dalian National Laboratory for Clean Energy, iChEM, Dalian Institute of Chemical Physics, Chinese Academy of Sciences, 116023 Dalian, People’s Republic of China

## Abstract

As the black cesium lead iodide (CsPbI_3_) tends to transit into a yellow δ-phase at ambient, it is imperative to develop a stabilized black phase for photovoltaic applications. Herein, we report a distorted black CsPbI_3_ film by exploiting the synergistic effect of hydroiodic acid (HI) and phenylethylammonium iodide (PEAI) additives. It is found that the HI induces formation of hydrogen lead iodide (HPbI_3+x_), an intermediate to the distorted black phase with appropriate band gap of 1.69 eV; while PEAI provides nucleation for optimized crystallization. More importantly, it stabilizes the distorted black phase by hindering phase transition via its steric effects. Upon optimization, we have attained solar cell efficiency as high as 15.07%. Specifically, the bare cell without any encapsulation shows negligible efficiency loss after 300 h of light soaking. The device keeps 92% of its initial cell efficiency after being stored for 2 months under ambient conditions.

## Abstract (web summary)

Black phase cesium lead iodide perovskite is regarded as a promising candidate for solar cells, but it easily transits to undesired yellow phase. Herein, Wang et al. stabilized the black phase using molecular additives to achieve device efficiency beyond 15% with high light soaking stability.

## Introduction

There have been incredible developments in organic-inorganic hybrid lead halide perovskite solar cells (PSCs) recently. In fact, their power conversion efficiency (PCE) has skyrocketed from 3.8 to 22.7% in just a few years^1–5^. Unfortunately, the hybrid perovskite suffers from unavoidable degradation because the hydrogen-bonding between its monovalent organic cation and octahedral PbI_2_ is very weak^6–10^. It degrades to PbI_2_ under common external stresses, such as electric fields^11,12^, moisture^13,14^, photo-oxidation^15,16^, and UV irradiation^17^. Substituting the organic cation by inorganic Cs^+^ to fabricate an all-inorganic perovskite is effective for improved stability under these common stress conditions^18,19^. Unfortunately, the most ideal black α-CsPbI_3_ (cubic phase) is thermodynamically less favorable. It spontaneously turns into an undesired δ-CsPbI_3_ orthorhombic phase under ambient conditions^20^.

There has been extensive research into the preparation of CsPbI_3_ solar cells. For example, Snaith et al.^20^ fabricated the first CsPbI_3_ solar cell via an HI additive with the highest PCE of only 2.9%. These trace amounts of HI were incorporated into the crystal lattice to form smaller grains with a distorted structure that stabilizes the cubic phase at room temperature. Luo et al.^21^ developed a new phase-transition scheme to fabricate α-CsPbI_3_ solar cells from Cs_4_PbI_6_ to increase the PCE to 4.13%. Further advancement has been proven difficult, it has taken more than a year for the PCE to be slowly improved to 4.68%^22,23^. Recently, vacuum-based vapor deposition was used to improve the PCE to 8.80%^24,25^. By accurately controlling the stoichiometric ratios of the precursors, Lin et al.^26^ and Troshin et al.^27^ increased PCEs to 9.40% and 10.5%, respectively. Unfortunately, all of these devices showed very poor stability, even the well-encapsulated cells lasted for only a few days in an inert atmosphere. Thus, it is critical to improve the stability while increasing the initial PCE.

The size-dependent phase diagrams suggest that the cubic phase becomes more stable when the nano-crystal size is decreased^28,29^. In particular, Luther et al. fabricatedα-CsPbI_3_ quantum dots (QDs) PSCs with a markedly improved PCE of 10.77%. Excitingly, this solar cell remained stable for 60 days in a dry environment with no loss of PCE^30^. Furthermore, high mobility QD films were fabricated by passivating surface of the α-CsPbI_3_ QDs using a halide salt with PCE of the corresponding solar cell being increased to 13.43%^31^. More recently, long-chain ammonium additives were found to have a profound impact on the resulted material structure and stability^32^. For instance, Zhao et al.^33^ found that the HPbI_3+*x*_ intermediate facilitates the formation of α-CsPbI_3_ films at lower temperature, while the ethylenediamine cations help stabilize the black α-CsPbI_3_ phase, making it possible to attain high cell efficiency of 11.8% with long-term stability for months. Kuang et al.^34^ introduced a bulky ammonium to form a stable two-dimensional CsPbI_3_-based PSC with a PCE of 4.84%. The initial PCE of This sample decreased slightly after being aged under ambient condition for over 30 days. Later, by using sulfobetaine zwitterions to stabilize the α-CsPbI_3_ film, Huang et al.^35^ developed PSCs with a PCE reaching up to 11.4% while it maintained 85% of its initial PCE after being stored in air exceeding 30 days.

The cubic phase can also be stabilized via partially substituting the I^−^ with Br^−^ ions^36^ to form CsPbBr_*x*_I_3−*x*_^37–40^. In particular, CsPbBr_3_, CsPbBr_2_I, CsPbI_2_Br, and CsPbI_2+*x*_Br_1-*x*_ have been used to fabricate PSCs with highly PCEs of 9.72%^41^, 8.02%^42^, 14.81%^43^, and 14.4%^44^, respectively, with good phase stability over several months. Unfortunately, the Br^−^ incorporation increases the band gap that is already too large for high-efficiency PSCs. It is also found that partial substitution of Cs^+^ and Pb^2+^ with smaller radius metal cations also improves the stability^45^. In fact, solar cells based on Cs_0.925_K_0.075_PbI_2_Br, CsPb_0.9_Sn_0.1_IBr_2_, CsPb_0.96_Bi_0.04_I_3_, and CsPb_0.98_Mn_0.02_I_2_Br also show respectable PCEs of 10.00^46^, 11.33^47^, 13.21^48^, and 13.47%^49^, respectively. In early 2013, based on theoretical calculations, Kanatzidis et al.^50^ proposed to use the distorted orthorhombic phase in perovskite solar cells. Later, Even et al.^51^ and Snaith et al.^52^ discovered that lower-symmetry (β-CsPbI_3_ and γ-CsPbI_3_) perovskites show much lower phase-transition temperature (260 and 175 °C, respectively) than the undistorted α-CsPbI_3_ (360 °C). These results suggest that the lower-symmetry perovskite orthorhombic black phase is beneficial to high stability devices^53^.

Herein, by mixing hydroiodic acid (HI) and phenylethylammonium iodine (PEAI) additives into the CsPbI_3_ precursor solution, we fabricate stabilized and distorted black phase-based CsPbI_3_ thin films with excellent crystallinity. To our surprise, the PCE of the CsPbI_3_ PSC is increased to 15.07%—the highest yet reported for this type of inorganic perovskite cell. The performance is also remarkably stable under ambient conditions.

## Results

**Fig. 1:**
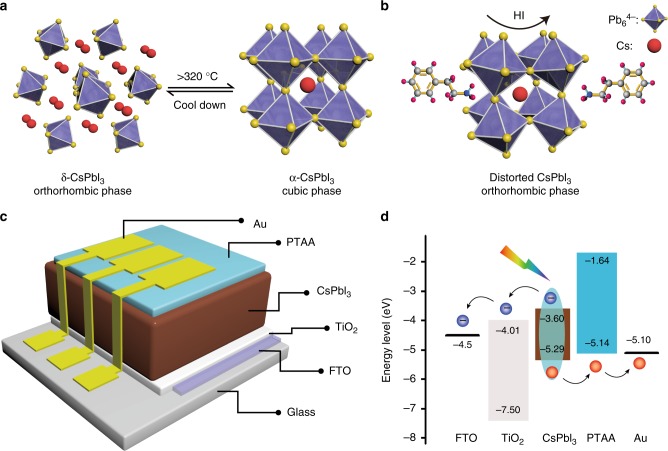
Structure and energy level: **a** Schematic of common CsPbI_3_ phases; **b** HI-induced and PEAI-stabilized distorted black CsPbI_3_ orthorhombic phase; **c** Schematic device structure; **d** energy level

Figure 1a shows the common yellow (δ-, Pnma) and black (α-, Pm3m) phases of the CsPbI_3_ films. According to the phase diagram, the desired α-CsPbI_3_ phase is stable only at high temperature (higher than 360 °C). The transition from the δ-phase to the α-phase indicates an obvious dynamic motion of the [PbI_6/2_]^−^ octahedral^51^. Upon cooling, it is first converted into the distorted black perovskite β-CsPbI_3_ (P4/mbm), γ-CsPbI_3_ (Pbnm), and then to yellow δ-CsPbI_3_ at 260, 175, and 25 °C, respectively. The four phases reported and their corresponding XRD patterns are given in Supplementary Fig. 1^51^. Note that the yellow δ phase consists of double-chains of non-corner-sharing [PbI_6/2_]^−^ octahedral, while the other three black phases show obviously corner-sharing [PbI_6/2_]^−^ octahedral^53,54^. Furthermore, the XRD peaks of the distorted phases (β-CsPbI_3_, γ-CsPbI_3_) are slightly splitted relative to the α-CsPbI_3_^55^.

We fabricated a distorted black phase CsPbI_3_ by using a small amount of HI and PEAI (Fig. 1b). Here, the HI is reported to firstly form the hydrogen lead iodide (HPbI_3+*x*_) intermediate with PbI_2_, which helps to grow the above-mentioned distorted phases (β-CsPbI_3_, γ-CsPbI_3_)^33^. The PEAI plays a dominant part in the nucleation and growth of the CsPbI_3_ film, in addition, it stabilizes the distorted black phase by hindering the phase transition through steric effects. For solar cell evaluation, a typical normal cell structure (glass substrate/fluorine-doped tin oxide (FTO)/TiO_2_/CsPbI_3_/Poly[bis(4-phenyl) (2,4,6-trimethylphenyl)amine] (PTAA)/Au) was used (Fig. 1c). Figure 1d shows the energy level band diagram. The energy levels of each layer were measured as reported previously^39^. The valence bands were obtained via ultraviolet photoelectron spectroscopy (UPS)^56,57^, and the conduction bands were estimated using the band gap derived from the optical absorption spectra.

A control film was synthesized using only PEAI without HI. It does not yield the dark perovskite phase even after annealing at 150 °C for 3 h. Therefore, we exclude it from the following discussion, and HI was used for all CsPbI_3_ films discussed below. The films prepared without the PEAI are termed as w/o PEAI, and the films with PEAI are labeled as w PEAI. In the present study, the amount of HI additive is further optimized to obtain larger grain size with desired film morphology. For this purpose, smaller amount of HI (100 μl) was used to carefully control the amount of HPbI_3+*x*_ formed to allow longer time of annealing at 150 °C to achieve the optimal crystal growth. Microscopic imaging was used to examine the CsPbI_3_ films at each stage of growth and treatment.

Supplementary Figs. 2a, 3a show optical microscopy (OM) images with lower resolution photos as insets. Supplementary Figs. 2b, 3b present high-resolution scanning electron microscope (SEM) images of the sample surfaces. Supplementary Fig. 3c shows atomic force microscope (AFM) images for the samples prepared with PEAI annealed at 150 °C for different length of time up to 10 h. Both films prepared with and without PEAI show small crystalline domain sizes of about 100 nm, smooth surfaces, and yellow in color before annealing. The statistics of crystal size distributions are given in Supplementary Fig. 4. It is clear that the longer the annealing time, the larger the grain size. In other words, the grains grow larger during the annealing process.

**Fig. 2:**
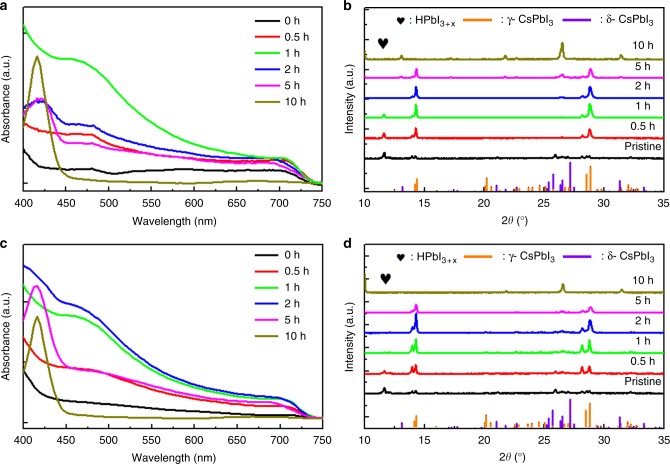
Performance of the fabricated films: Comparison of the CsPbI_3_ films without and with PEAI annealed at 150 °C for various times: **a**, **c** optical absorption spectra; **b**, **d** XRD patterns

We observed that the CsPbI_3_ film is formed rapidly upon the deposition of the precursor solution. With increasing the annealing time, the as-deposited yellow film quickly turns into black, with increased crystalline domain size, as shown in Supplementary Fig. 4. Without the PEAI additive, the blackest uniform sample was obtained after annealing for only 1 h. Further heat treatment produces isolated yellow spots as shown in Supplementary Fig. 2a, suggesting that part of the sample has gone through a phase transition from the black phase to the yellow δ phase. As the process continues, the yellow spots became larger and larger, until they finally turn into a complete yellow film, identified to be the δ-CsPbI_3_ phase by X-ray diffraction (XRD) shown in Fig. 2. In comparison, when PEAI was used, it took up to 2 h of annealing before the black uniform film was completely formed, indicating that the CsPbI_3_ formation process is slower. Further heating beyond 2 h would result in yellow pinholes.

To gain insights into the phase transition mechanism, we measured optical absorption, XRD, and X-ray photoelectron spectroscopy (XPS). Figure 2a, c compares the absorption spectra of the CsPbI_3_ films fabricated at different annealing time at 150 °C. It shows that the highest absorbance for the CsPbI_3_ films without and with PEAI occurred after annealing for 1 and 2 h, respectively. Further annealing leads to decreased absorption intensities for the black phase, while the absorption peak at 420 nm for the δ-CsPbI_3_ phase increases, suggesting that the black CsPbI_3_ are partially transitioned into the yellow δ-CsPbI_3_ phase. These results are consistent with the optical photographs. On the other hand, the surface elemental distributions of the films as analyzed using the XPS spectra (Supplementary Fig. 5) do not show any change with annealing time, indicating that the change in optical absorbance is due to evolution of the crystalline structure.

The XRD patterns were compared to the reference (Supplementary Fig. 1). There is no obvious difference between the films with and without PEAI before the annealing treatment. After annealing the sample for 1 h, the CsPbI_3_ film prepared without PEAI shows optimum crystallization with surplus HPbI_3+*x*_ (Fig. 2b)^58^. As the annealing time is prolonged, the intensities of the distorted-phase-based CsPbI_3_ peaks decrease, and the δ-CsPbI_3_ phase peaks start to appear. Finally, the film is transitioned completely into the δ-CsPbI_3_ phase. In comparison, the CsPbI_3_ formed with PEAI (Fig. 2d) shows well defined peaks of distorted CsPbI_3_ phase after annealing for 2 h, demonstrating that the PEAI did not alter the crystalline structure of the perovskite film. Furthermore, careful examination shows that the distorted black phase transited from β to the γ phase during the annealing process, as identified by the XRD peaks slowly shifting from 14.2° to 14.3° (Supplementary Fig. 6).

The effect of the PEAI was further studied by comparing the optimized distorted black phase-based CsPbI_3_ films deposited without and with PEAI. Cross-sectional SEM images (Supplementary Fig. 7a), UV–vis absorption spectra (Supplementary Fig. 7b), and photoluminescence (PL) spectra (Supplementary Fig. 7c) show that there is no obvious difference between the films prepared without and with the PEAI doping after they are annealed. Both films show same band gap of 1.69 eV, slightly lower than the value of 1.73 eV as reported in literature^59^. X-ray photoelectron spectroscopy (XPS, Supplementary Fig. 7d) and the vertical composition profiles from the surface (Supplementary Fig. 8) show clear characteristic peaks assigned to Cs 3*d*, Pb 4*f*, and I 3*d*, confirming that the added PEAI did not affect the composition of the films. Meanwhile, the other peaks show no change or shifting after adding the PEAI, indicating that the PEAI molecules bond to the surface of the crystal grain units only for the PEA^+^ are too large to be inset into the CsPbI_3_ cages^53^. Furthermore, the XRD results in Supplementary Fig. 7e show that the crystallization quality obviously improved by the PEAI additive. The orientation was obviously optimized as the XRD does not show peaks for (111), (020), and (120), likely because the PEA^+^ ions act as steric hindrance during the CsPbI_3_ crystal growth, leading to preferred orientation for (001), (110), (002), and (220). The energy-dispersive X-ray (EDX) analysis and contact angle measurements, shown in Supplementary Fig. 9 further confirm that PEAI is distributed uniformly in the grain boundary and surface.

**Fig. 3:**
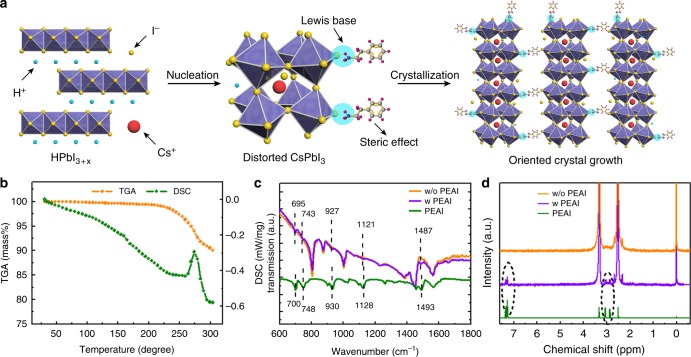
Mechanism of HI/PEAI-induced phase stability: **a** Schematic for HI/PEAI additive-induced CsPbI_3_ crystal growth. **b** TGA and DSC spectra for the CsPbI_3_ film with PEAI. **c** FTIR spectra, and **d** ^1^H liquid-state NMR spectra for the related films

Figure 3a presents a schematic diagram for the HI and PEAI additive-induced CsPbI_3_ crystalline growth. From the results, we infer that: (1) initially, HI reaction with PbI_2_ as intermediate HPbI_3+*x*_, and it creates distorted black phase-based CsPbI_3_ after combined with CsI during annealing at 150 °C. The results are consistent with previous studies^58,60^. (2) PEAI does not cause any change in the chemical composition of the final crystal structure, and likely plays only significant role in the nucleation and growth of CsPbI_3_ films that lead to oriented crystal growth. (3) the PEAI acts as a blocking element to hinder the phase transition by bonding to the crystal units and through the steric effect. Supplementary Fig. 10 shows that the film without PEAI degraded within a few days for the black phase to transition to the yellow phase. Moreover, the PEAI based CsPbI_3_ film can retain the black color even after being heated at 80 °C for 120 h, while the reference film shows yellow spots in only 40 h (Supplementary Fig. 11). In summary, the distorted black phase-based CsPbI_3_ films obtained using the HI and PEAI additives show excellent properties, including large grain size, better thermal stability and lower band gap.

The thermal stability of the present CsPbI_3_ system was further confirmed by thermogravimetric analysis (TGA) and differential scanning calorimeter (DSC), as shown in Fig. 3b. When the sample is heated in nitrogen, no mass loss was observed until it gets from room temperature to 225 °C, at which point the film starts to decompose. The addition of PEAI is not found to have any detectable effect on thermal stability of the perovskite film (Supplementary Fig. 12) except that it starts to evaporate or decompose at about 300 °C. To confirm that the PEAI is indeed incorporated into the film, we examined the films (PEAI, CsPbI_3_, and CsPbI_3_ film with PEAI) using the Fourier transform infrared spectroscopy (FTIR, Fig. 3c). Clearly, the spectrum of the CsPbI_3_ film with PEAI shows absorption bands centered at 695, 743, 927, and 1121 cm^−1^ that are signatures of the benzene ring in the PEA^+^. Moreover, a peak at 1487 cm^−1^ is also resolved, ascribed to the N-H scissor bending in the PEA^+^^37^. All these results attest the existence of PEA^+^ in the CsPbI_3_ film. Meanwhile, careful comparison of the FTIR spectrum with that of the pure PEAI show that, all above peaks are shifted to smaller wavenumbers, demonstrating strong interaction between the functional groups of PEA^+^ and CsPbI_3_^61^. We believe that one major reason is the chemical bonding between the PEAI and the CsPbI_3_^33,62^. To further prove that significant amount of PEA^+^ is in the film, the nuclear magnetic resonance (NMR) measurement was conducted on the dissolved sample in deuterated DMSO-d_6_ solution (Fig. 3d). The pure PEAI gives two peaks at *δ* = 7.3 and 3 ppm, attributed to the benzene ring and its branched chain group, respectively. For the CsPbI_3_ film prepared with PEAI, the above two peaks shifted to downfield by Δ*δ* ≈ 0.1 ppm, indicating strong interaction between the PEA^+^ and CsPbI_3_^63^.

**Fig. 4:**
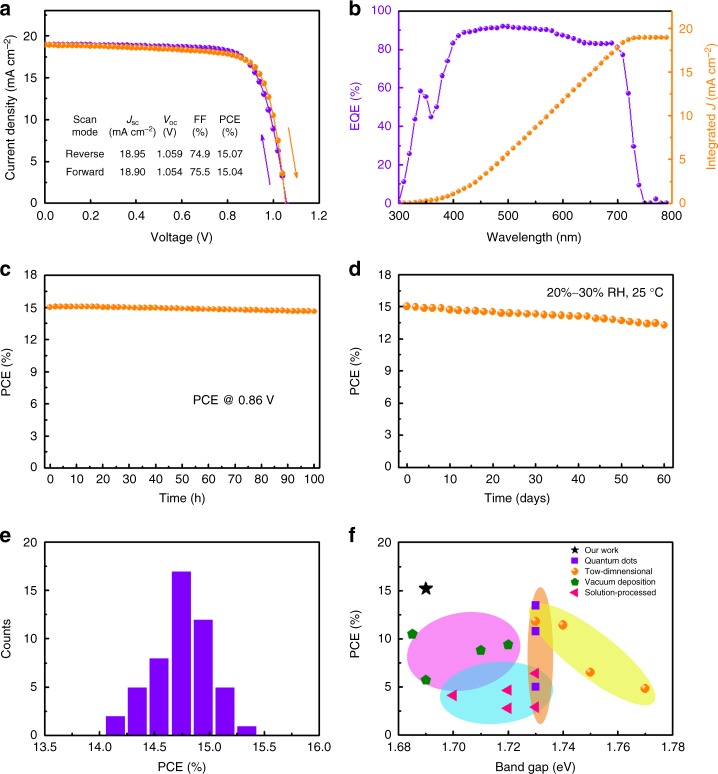
Performance of the best device with PEAI: **a** *J*–*V* characteristics, **b** EQE, **c** steady-state measurement of the PCE, **d** long-term stability of the best-performing device, **e** histogram of device efficiency distribution over 50 cells, and **f** PCE distribution of CsPbI_3_ PSCs as a function of band gap relative to published papers

Finally, the films based PSCs were fabricated to evaluate their photovoltaic performance. The current density (*J*)–voltage (*V*) curves and photovoltaic parameters are presented in Supplementary Fig. 13 and Supplementary Table 1. Clearly, the optimal annealing conditions for the cells agree with the values reported above. The results for the CsPbI_3_ films (w/o PEAI) are shown in Supplementary Fig. 14. It shows a remarkable cell PCE of 11.50%, with open-circuit voltage (*V*_OC_) of 0.991 V, short-circuit current density (*J*_SC_) of 16.94 mA cm^−2^, and fill factor (FF) of 68.6%. The cell performance improves significantly when PEAI is introduced. When the film is optimized, the *V*_OC_, *J*_SC_, FF, and PCE are 1.059 V, 18.95 mA cm^−2^, 75.1, and 15.07%, respectively. Figure 4a presents typical *J*–*V* curves measured using both reverse and forward scan directions with the key parameters summarized in the inset. It is clear that the *J*–*V* curves measured using both scan directions overlap well with each other. In other words, the hysteresis of the present devices is negligible. It is known that the *V*_OC_ deficit which defined by *E*_g_/*q*-*V*_OC_ limits the PCE efficiencies^64^. Here, *E*_g_ is the optical band gap and *q* electron charge. The *V*_OC_ deficit for our fabricated CsPbI_3_ PSCs is 0.631 eV, larger than the best reported for organic–inorganic hybrid solar cells^65^, indicating to further increase device performance, reducing defect density in CsPbI_3_ layer or the interfaces are urgently expected.

The incident photon-to-electron conversion efficiency (IPCE) shows an integrated *J*_SC_ value of 18.86 mA cm^−2^ (Fig. 4b), which matches well with the value from the *J*–*V* curve. Figure 4c presents the steady PCE versus time while being biased at the maximum power point (0.86 V); the champion device exhibits a very stable PCE and less than 3% efficiency loss after 100 h of measurements. Long-term stability is critical for the perovskite solar cells. Figure 4d shows that the device maintains 92% of its best PCE value after storage in air (relative humidity of 20 to 30% at about 25 °C) for over 60 days without any encapsulation. To test the light stability, the bare device without any encapsulation was placed under constant AM 1.5G illumination in a nitrogen filled glovebox for more than 300 h. The device had negligible efficiency loss (Supplementary Fig. 15) demonstrating its steadiness to the light irradiation.

The PCE distribution for a batch of 50 solar cells is also shown in Fig. 4e. The data demonstrate that the devices are highly reproducible with an average PCE of 14.6%. The best CsPbI_3_ film-based device prepared here without PEAI demonstrated poor stability with PCE degradation to 0.65% after storage in ambient environment for 8 days (Supplementary Fig. 14). We also compared the results to those of other reported CsPbI_3_ PSCs. Figure 4f plots PCE versus band gap for the previously reported high PCE PSCs formed using various processing methods^18–33^. Our device with a distorted black phase-based CsPbI_3_ achieved the best PCE.

## Discussion

In conclusion, we report an all-inorganic perovskite film based on distorted black phase CsPbI_3_. By introducing HI and PEAI additives, a well crystallized black phase-based CsPbI_3_ film is produced. The optimized PSC shows a record PCE of 15.07% with remarkable stability. It is expected that these distorted black phase CsPbI_3_ perovskites have significant potential applications in other optoelectronic devices in the future.

## Methods

### Precursor solutions preparation

The CsPbI_3_ precursor solution used HPbI_3+x_ (0.88 g) and CsI (0.36 g) dissolved in 2 ml DMF/DMSO (v/v 9:1) in N_2_ atmosphere in a glovebox under active stirring for 24 h. The CsPbI_3_-PEAI precursor solution with 100 μl PEAI DMF solution (100 mg ml^−1^) was then added to the above CsPbI_3_ precursor solution under active stirring for 6 h. A HTL solution was prepared by dissolving PTAA (36 mg), a sulfonyl imide (Li-TFSI, 22 μl, 520 mg Li-TFSI in 1 ml acetonitrile), and tert-butylpyridine (TBP, 36 μl) in 1 ml of CB solution.

### Device fabrication

A 25 × 25 mm^2^ piece of clean FTO substrate was surface treated using an O_2_ plasma, and then the compact TiO_2_ layer coated on the FTO was formed by immersing FTO in a 40 mM TiCl_4_ aqueous solution at 70 °C. Then, the CsPbI_3_ layer was fabricated via spin-coating of the solution onto the substrate. Finally, the films were annealed at 150 °C for various time to form the films. PTAA layer was prepared by spin-coating the related precursor onto the CsPbI_3_ film. A 70-nm-thick gold electrode was then thermally evaporated onto the HTL-coated film.

### Characterization

The film surface morphology and cross-sectional images were characterized by FESEM and EDS (Jeol SU-8020). The AFM images were acquired using a Veeco NanoScope IV with a silicon cantilever. Contact angles were measured on an OCA20 instrument (Data-physics). Absorbance spectra were collected using a Shimadzu UV-3600 double beam spectrometer. The PL spectra were measured using a PicoQuant FluoTime 300. The source light was a xenon short arc lamp. XRD patterns of the samples were measured using a Bruker D8 GADDS Diffractometer with the Cu Kα line. The XPS measurements were performed in a VG ESCALAB MK2 system with monochromatized Al Kα radiation. TGA/DSC was performed using a TGA Q50 (TA Instruments) and DSC Q2000 (TA Instruments), respectively. FTIR spectra observations of samples were carried out on a Bruker EQUINX55 spectrometer. Liquid-state NMR spectra were conducted on a VNMRS 600 instrument. The device active area was 0.1156 cm^2^ (3.4 mm × 3.4 mm), and a mask with an aperture area of 0.09028 cm^2^ was used to prevent any scattered light or light piping to contribute to the photocurrent. The light source was performed via the solar simulator (SS-F5-3A, Enlitech) along with AM 1.5 G spectra whose intensity was calibrated by the certified standard silicon solar cell (SRC-2020, Enlitech) at 100 mW cm^−2^. *J–V* curves were measured using reverse scan mode (from *V*_OC_ to *I*_SC_) and forward scan mode (from *I*_SC_ to *V*_OC_) with a scan rate of 30 mV s^−1^. The spectral response was taken by an EQE measurement system (QE-R, Enlitech). The long-term stability was measured after storage in air (relative humidity of 20 to 30% at 25 °C) over 60 days without any encapsulation. To test the device light stability, we put the unencapsulated device in a nitrogen glovebox under constant AM 1.5G illumination.

## Electronic supplementary material

Supplementary Information

## Data Availability

The data that support the findings of this study are available from the corresponding author upon reasonable request.
