# Publisher Correction: All-inorganic cesium lead iodide perovskite solar cells with stabilized efficiency beyond 15%

**DOI:** 10.1038/s41467-018-07485-3

**Published:** 2018-11-19

**Authors:** Kang Wang, Zhiwen Jin, Lei Liang, Hui Bian, Dongliang Bai, Haoran Wang, Jingru Zhang, Qian Wang, Shengzhong Liu

**Affiliations:** 1 Key Laboratory of Applied Surface and Colloid Chemistry, Ministry of Education, Shaanxi Key Laboratory for Advanced Energy Devices, Shaanxi Engineering Lab for Advanced Energy Technology, School of Materials Science & Engineering, Shaanxi Normal University, 710119 Xi’an, People’s Republic of China; 2 School of Physical Science and Technology & Key Laboratory for Magnetism and Magnetic Materials of MoE, Lanzhou University, Lanzhou, 730000 People’s Republic of China; 3 Dalian National Laboratory for Clean Energy, iChEM, Dalian Institute of Chemical Physics, Chinese Academy of Sciences, 116023 Dalian, People’s Republic of China

Correction to: *Nature Communications*, 10.1038/s41467-018-06915-6; published online 31 October 2018

In the original version of this Article, the author name ‘Shengzhong Liu’ was incorrectly given as ‘Liu Shengzhong’. This has been corrected in both the PDF and HTML versions of the Article.

