# Supplementary material for: All-inorganic cesium lead iodide perovskite solar cells with stabilized efficiency beyond 15%
Source: Nat Commun. 2018 Oct 31;9:4544. doi: 10.1038/s41467-018-06915-6 (PMC6208436; doi:10.1038/s41467-018-06915-6)
Supplement: Supplementary file 1 — Supplementary Information [file 41467_2018_6915_MOESM1_ESM.pdf]

Supporting Information

All-Inorganic cesium lead iodide perovskite solar cells with stabilized efficiency beyond 15%

Kang Wang[1], Zhiwen Jin[1,*], Lei Liang[1], Hui Bian[1], Dongliang Bai[1], Haoran Wang[1], Jingru Zhang[1], Qian Wang[1,*], and Shengzhong (Frank) Liu[1,2,*]

[1]Key Laboratory of Applied Surface and Colloid Chemistry, Ministry of Education; Shaanxi Key Laboratory for Advanced Energy Devices; Shaanxi Engineering Lab for Advanced Energy Technology; School of Materials Science & Engineering, Shaanxi Normal University, Xi'an, 710119, P. R. China.
E-mail: jinzhiwen@snnu.edu.cn, wangqian16@snnu.edu.cn

[2]Dalian National Laboratory for Clean Energy; iChEM, Dalian Institute of Chemical Physics, Chinese Academy of Sciences, Dalian, 116023, P. R. China
E-mail: szliu@dicp.ac.cn

Keywords: $CsPbI_3$, perovskite, solar cells, stability, efficiency

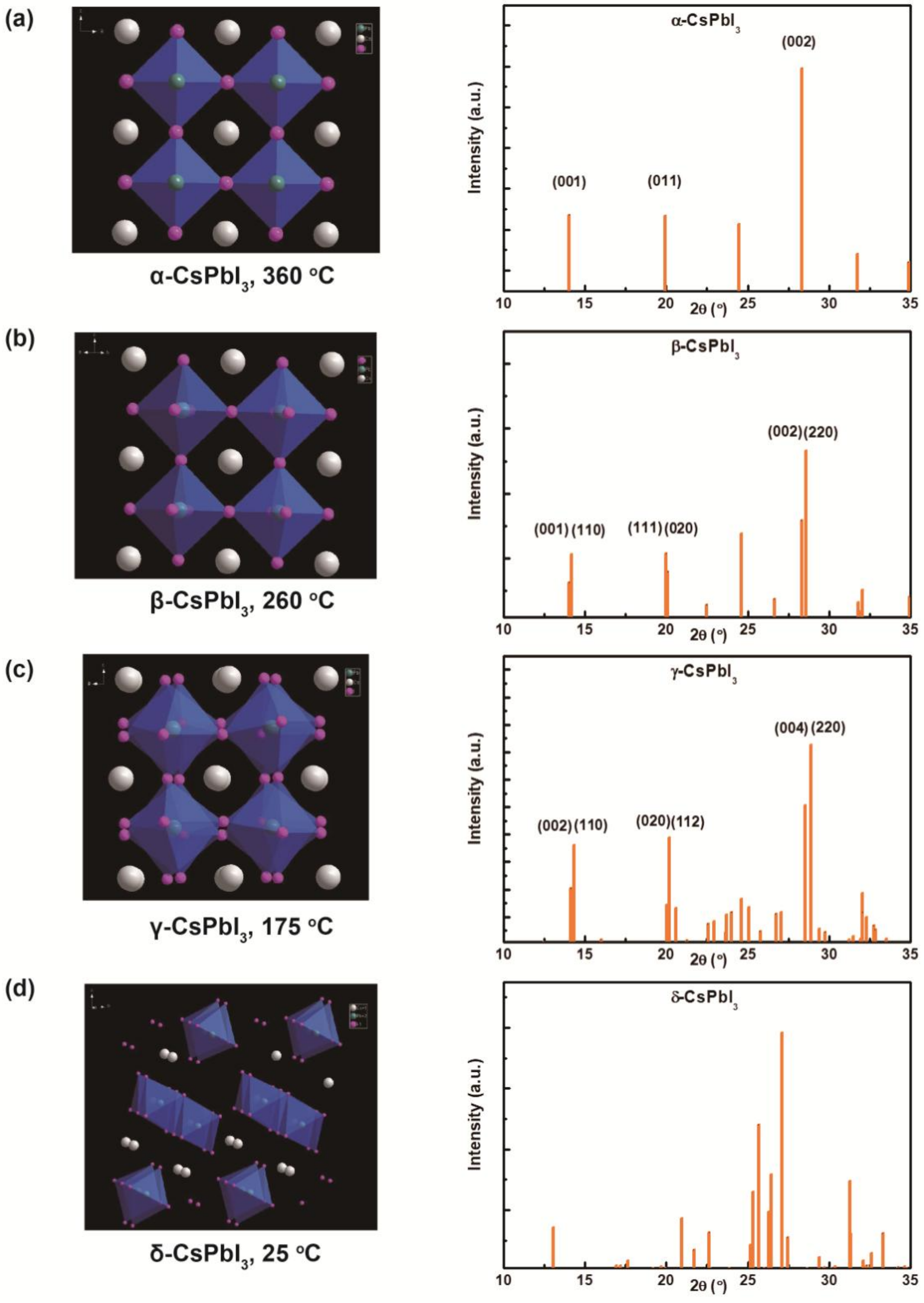

Supplementary Fig. 1 Diagrammatic structure and XRD patterns of $CsPbI_3$ phases: (a) α-$CsPbI_3$, (b) β-$CsPbI_3$, (c) γ-$CsPbI_3$, and (d) δ-$CsPbI_3$.

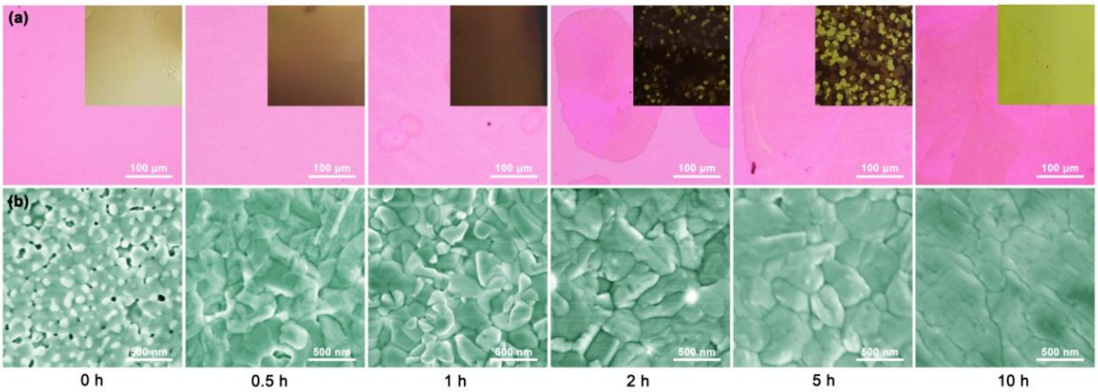

Supplementary Fig. 2 (a) Optical images and (b) SEM images of the $CsPbI_3$ films without PEAI after being annealed at 150 ℃ for various times.

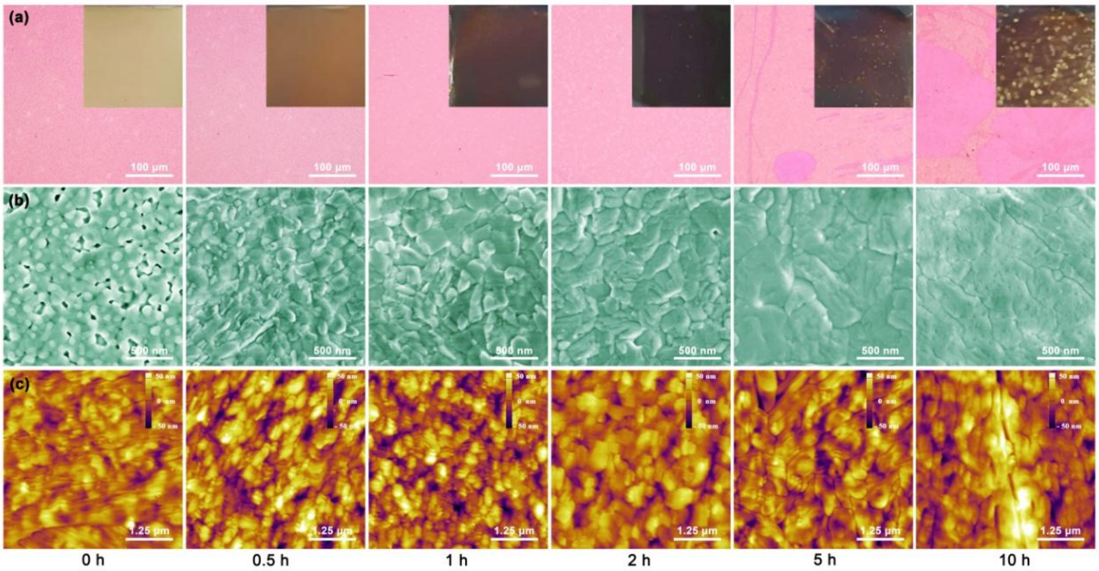

Supplementary Fig. 3 (a) Optical images, (b) SEM images, and (c) AFM images of the $CsPbI_3$ films with PEAI after being annealed at 150 ℃ for various times.

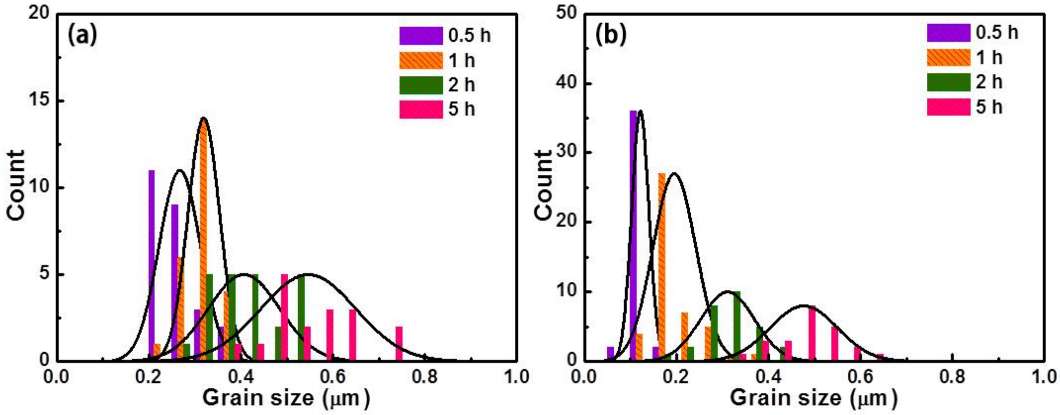

Supplementary Fig. 4 Histograms of the $CsPbI_3$ grain sizes obtained after annealing at 150 ℃ for various times: (a) without and (b) with PEAI additives.

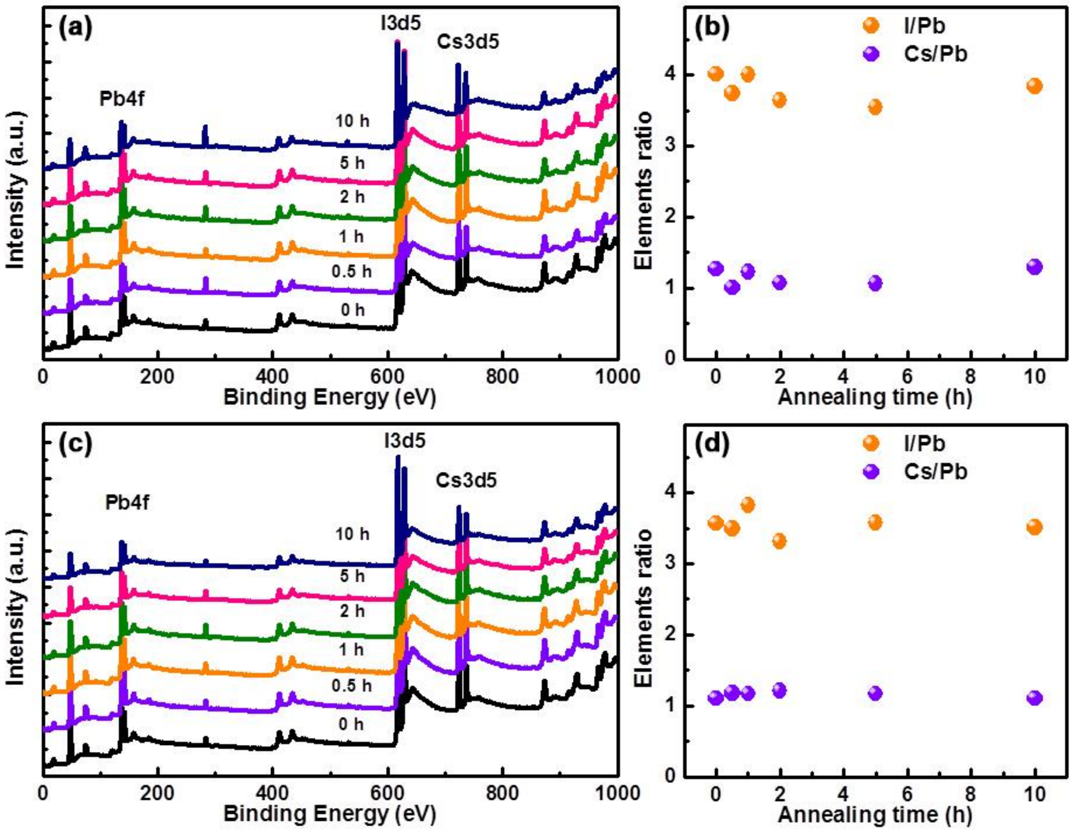

Supplementary Fig. 5 $CsPbI_3$ films obtained with (a and b) and without (c and d) PEAI after annealing at 150 ℃ for various times: (a) and (c) XPS spectra; (b) and (d) the elemental ratios.

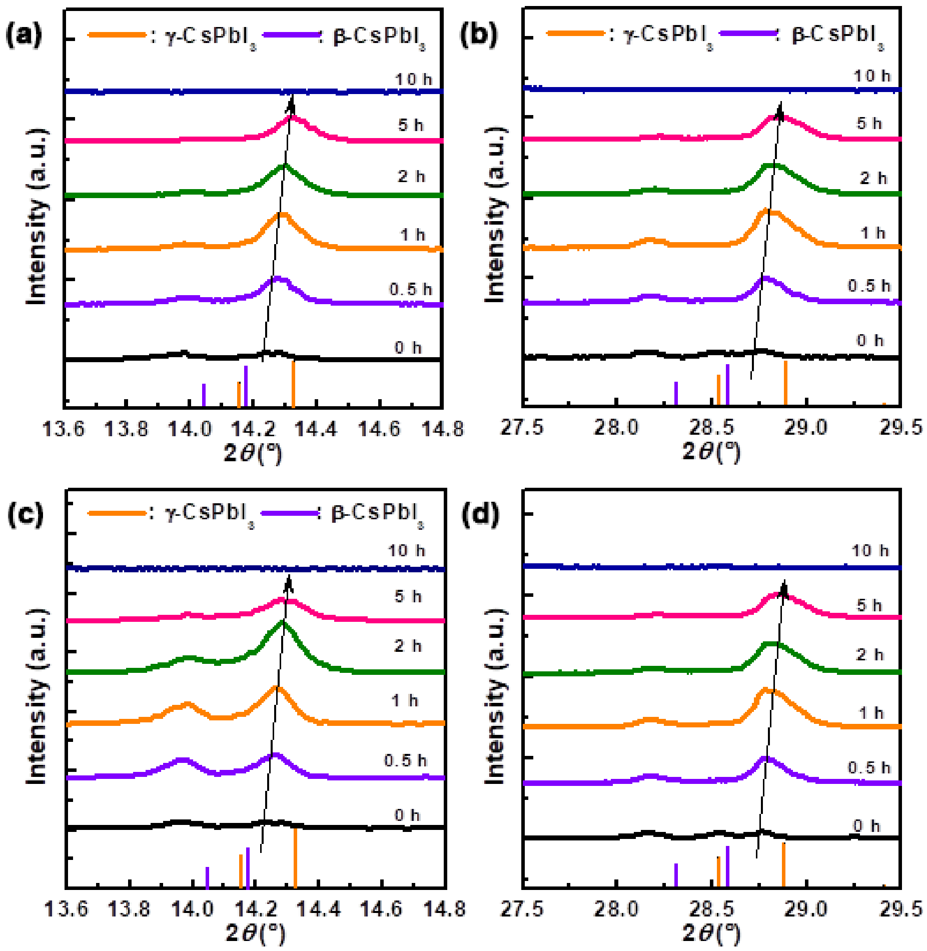

Supplementary Fig. 6 Enlarged XRD patterns of **Fig. 2**: (a) and (b) for $CsPbI_3$ films without PEAI; (c) and (d) for $CsPbI_3$ films with PEAI.

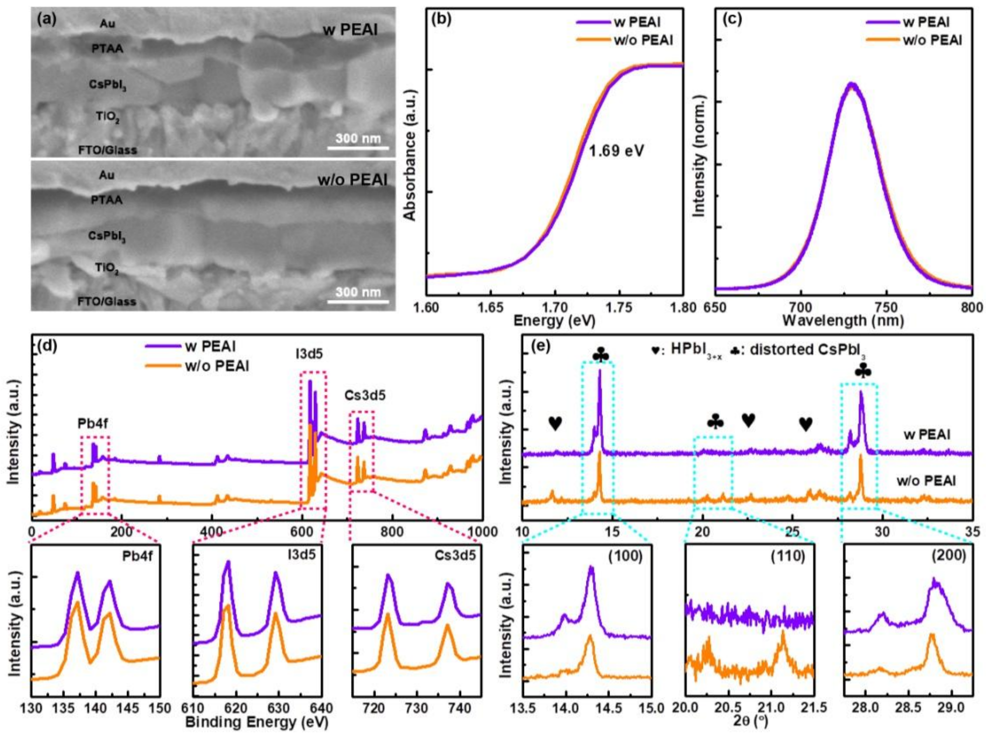

Supplementary Fig. 7 (a) Cross-sectional SEM images, (b) optical absorption spectra, (c) PL spectra, (d) XPS spectra, and (e) XRD patterns of the optimized $CsPbI_3$ films with and without PEAI.

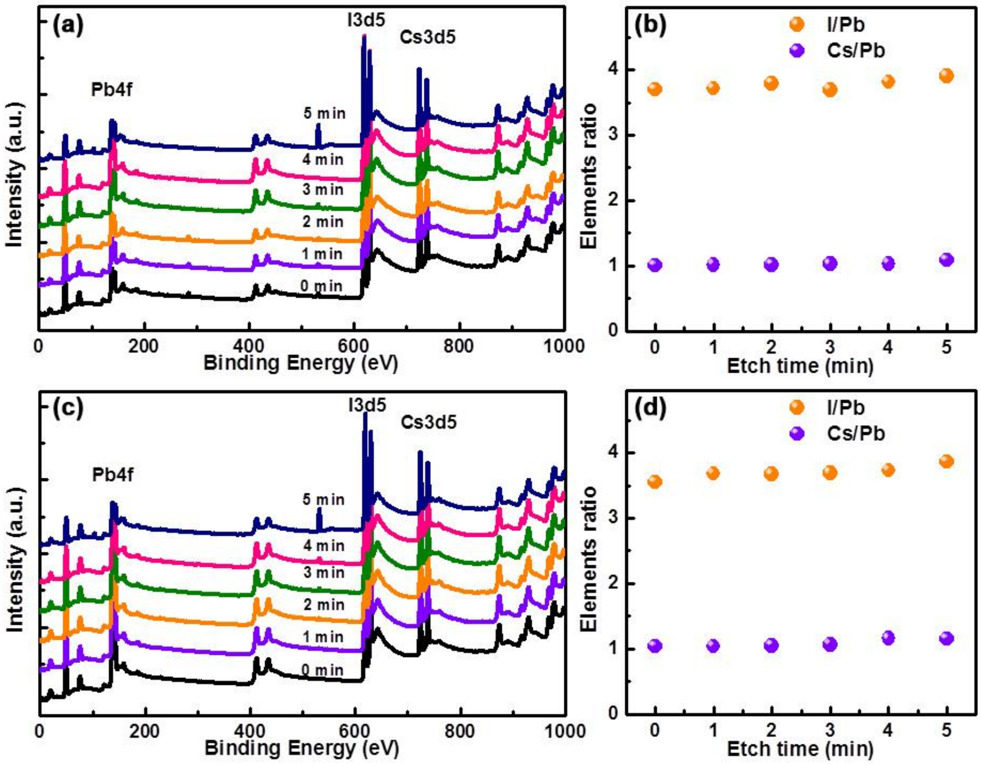

Supplementary Fig. 8 Vertical composition profiles from the surface (deduced via etching and XPS characterization) for the optimized $CsPbI_3$ films with (a and b) and without (c and d) PEAI: (a) and (c) for XPS spectra, and (b) and (d) for the elemental ratio.

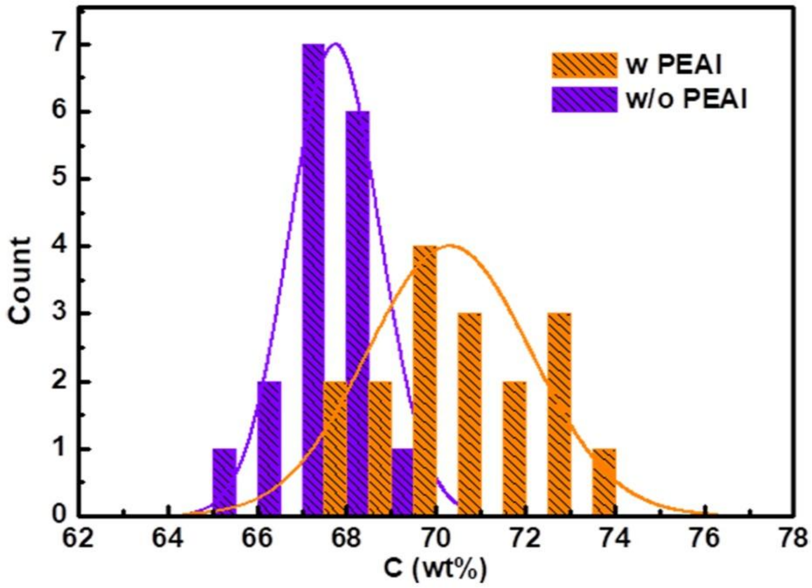

Supplementary Fig. 9 The EDX elemental distribution of C element at different parts of $CsPbI_3$ films with and without PEAI.

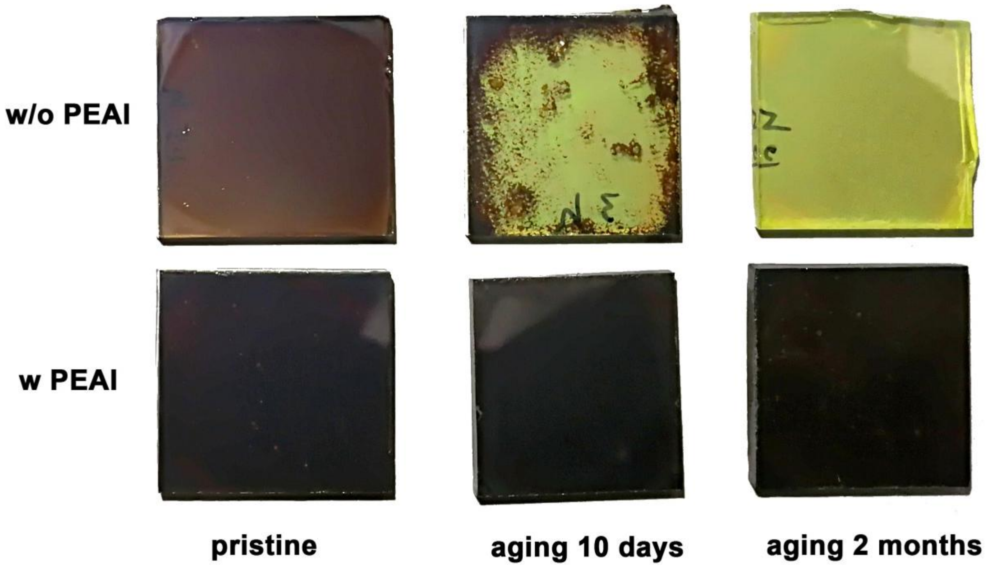

Supplementary Fig. 10 Photos of the $CsPbI_3$ films with and without PEAI stored in the ambient environment without encapsulation (25 ℃ and RH in the range of 20% to 30%) at different times.

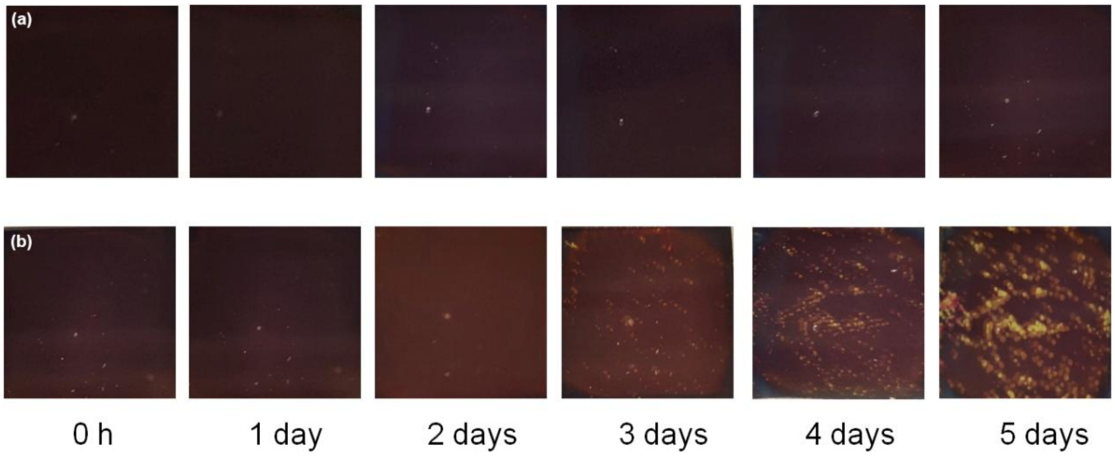

Supplementary Fig. 11 Photos of the $CsPbI_3$ films (a) with and (b) without PEAI annealing at 80 ℃ for different days.

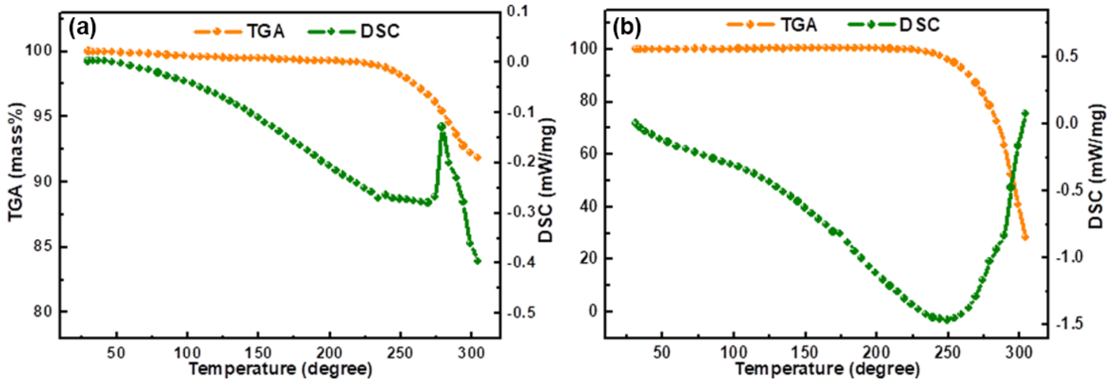

Supplementary Fig. 12 TGA and DSC spectra for (a) $CsPbI_3$ film and (b) PEAI film.

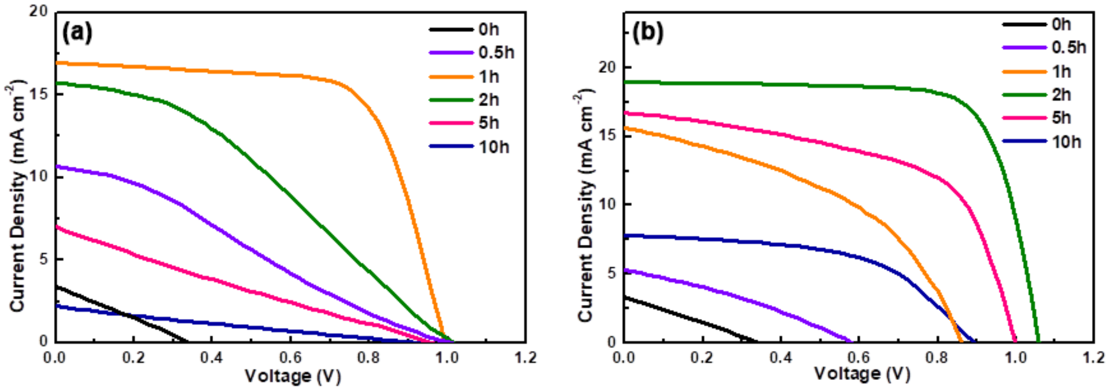

Supplementary Fig. 13 J-V characteristics of the PSCs based on $CsPbI_3$ films (a) without and (b) with PEAI after annealing at 150 ℃ for various times.

Supplementary Table 1. Comparison of the parameters of the PSCs based on different $CsPbI_3$ films (extracted from **Supplementary Fig. 13)**.

	Annealing time (h)	J_{SC} (mA cm^{-2})	V_{OC} (V)	FF (%)	PCE (%)		Annealing time (h)	J_{SC} (mA cm^{-2})	V_{OC} (V)	FF (%)	PCE (%)
**w/o PEAI**	0	3.39	0.333	26.6	0.30	**w PEAI**	0	3.39	0.342	26.7	0.31
	0.5	7.00	1.004	22.2	1.56		0.5	5.30	0.576	32.4	0.99
	1	16.94	0.991	68.6	11.50		1	15.62	0.858	44.0	5.90
	2	15.73	1.011	35.4	5.52		2	18.95	1.059	75.1	15.07
	5	10.64	0.949	28.4	2.87		5	16.75	0.998	57.5	9.61
	10	2.20	0.908	23.5	0.47		10	7.87	0.894	53.0	3.73

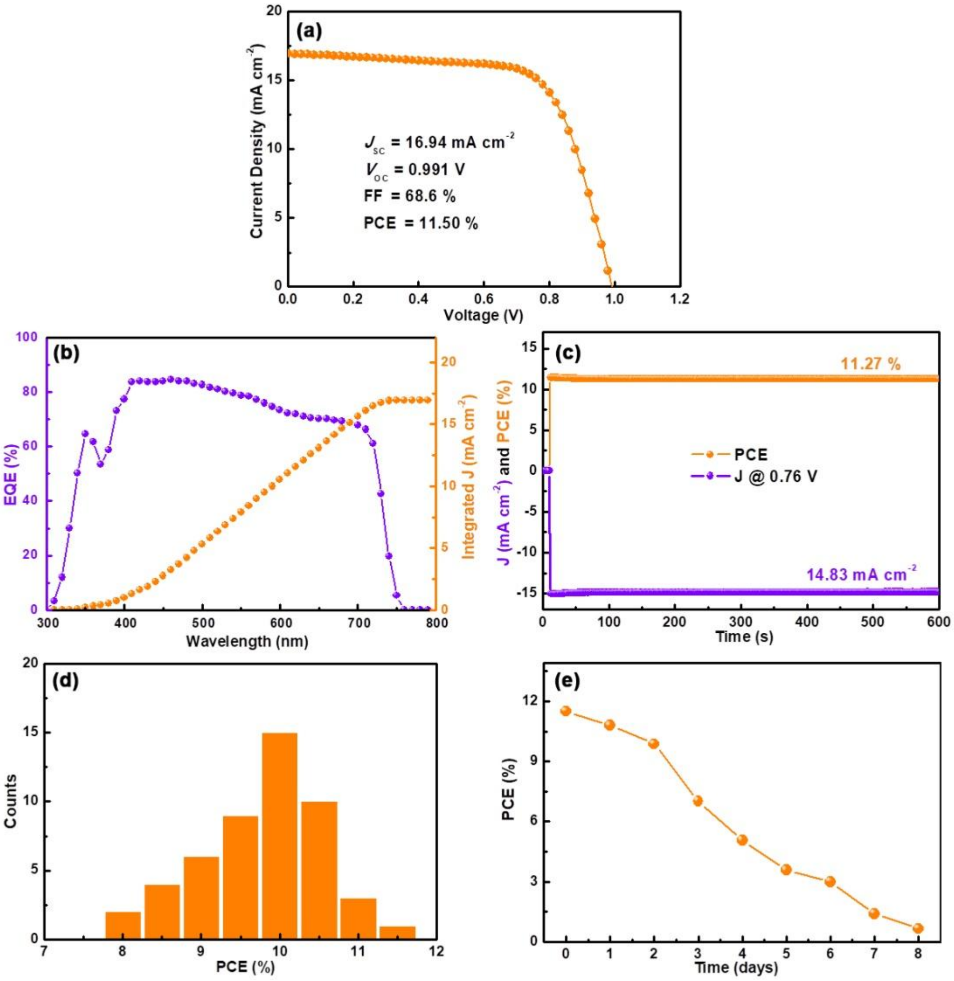

Supplementary Fig. 14 Performance of the best device without PEAI: (a) *J-V* characteristics, (b) EQE and the integrated product of the EQE curve with the AM 1.5 G photon flux, (c) steady-state measurements of the photocurrent and PCE, (d) histogram of device efficiency distribution of 50 cells, and (e) long-term stability of the best-performing device kept in an ambient environment without encapsulation (25 ℃ and RH in the range of 20% to 30%).

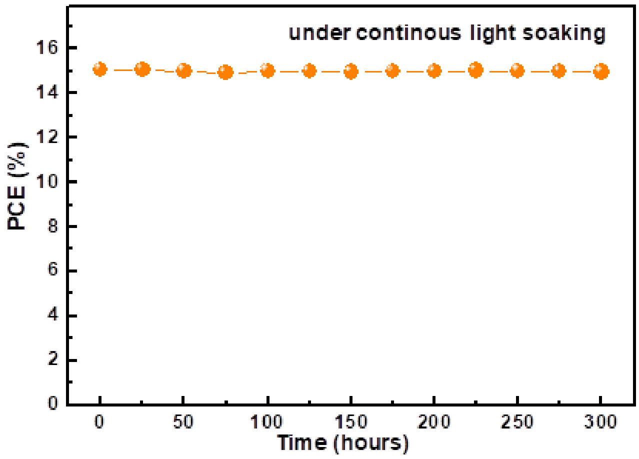

Supplementary Fig. 15 Continuous irradiation for over 300 h of the unencapsulated champion device in a nitrogen glovebox under constant AM 1.5 G illumination.
